# Construction of immune‐related risk signature for renal papillary cell carcinoma

**DOI:** 10.1002/cam4.1905

**Published:** 2018-12-05

**Authors:** Zhongyu Wang, Qian Song, Zuyi Yang, Jianing Chen, Jun Shang, Wen Ju

**Affiliations:** ^1^ Department of Urology, Union Hospital, Tongji Medical College Huazhong University of Science and Technology Wuhan Hubei Province China; ^2^ Department of Medical Oncology Fudan University Shanghai Cancer Center Shanghai China; ^3^ Department of Oncology, Shanghai Medical College Fudan University Shanghai China; ^4^ Department of Hematology The First Affiliated Hospital of Soochow University Suzhou China; ^5^ Department of Thoracic and Cardiovascular Surgery The First Affiliated Hospital of Guangxi Medical University Nanning China

**Keywords:** elastic net, papillary renal cell cancer, prognosis, tumor immunology

## Abstract

The kidney renal papillary cell carcinoma (KIRP) is a relatively rare type of kidney cancer. There has been no investigation to find a robust signature to predict the survival outcome of KIRP patients in the aspect of tumor immunology. In this study, 285 KIRP samples from The Cancer Genome Atlas (TCGA) were randomly divided into training and testing set. A total of 1534 immune‐related genes from The Immunology Database and Analysis Portal (ImmPort) were used as candidates to construct the signature. Using univariate Cox analysis, we evaluated the relationship between overall survival and immune‐related genes expression and found 272 immune‐related genes with predicting prognostic ability. In order to construct an efficient predictive model, the Cox proportional hazards model with an elastic‐net penalty was used and identified 23 groups after 1000 iterations. As a result, 15‐genes model showing more stable than other gene groups was chosen to construct our immune‐related risk signature. In line with our expectations, the signature can independently predict the survival outcome of KIRP patients. Patients with high‐immune risk were found correlated with advanced stage. We also found that the high‐immune risk patients with higher PBRM1 and SETD2 mutations, increasing chromosomal instability, together with the gene set enrichment analysis (GSEA) results showing intensive connection of our signature with immune pathways. In conclusion, our study constructs a robust 15‐gene signature for predicting KIRP patients’ survival outcome on the basis of tumor immune environment and may provide possible relationship between prognosis and immune‐related biological function.

## 1 INTRODUCTION

Kidney cancer has gradually become a commonly diagnosed cancer type worldwide both in men and in women. Globally, there are approximately 380 000 people who are diagnosed with kidney cancer and 143 000 patients who die from kidney cancer per year.^1^ Kidney renal papillary cell carcinoma (KIRP) comprises 15% to 20% of kidney cancers, which stems from the proximal nephron, the same origin with clear cell type.^2^ However, KIRP is considered as a more heterogeneous disease in the aspects of disease progression and patients’ survival outcomes.^3^ Targeted therapies such as the mTOR inhibitor and VEGF receptor inhibitor just provide modest benefit for those with metastasis.^4^, ^5^ In addition, due to the limited number of KIRP cases, KIRP patients are often excluded from molecular investigations and randomized clinical trials for kidney cancer.^6^ Therefore, there is still lack of solid data for further investigation the molecular profiling of KIRP, which may unearth novel targets for diagnosis, treatment, and prognosis of KIRP.

Immunotherapies such as programmed death‐1 (PD‐1)/programmed death ligand 1 (PD‐L1) inhibitors or tumor vaccine are becoming favorable novel treating approach for KIRP. The PD‐1/PD‐L1 blockade demonstrated modest antitumor activity in KIRP patients, with the response rate being approximately 30%,^7^ which are much higher than that of clear cell type. Besides, other studies found that a tumor vaccine called TroVax® which expressed the 5 T4 tumor‐associated antigen exhibited robust efficacy and safety in renal cell cancer patients including KIRP patients in clinical trials.^8^, ^9^ These shed lights on the importance of tumor immune environment characterization and the treatment efficacy of immunotherapy for KIRP.^11^, ^12^ Studies have shown that the components of tumor immune environment are correlated with patients’ survival outcomes. Stéphane Chevrier et al^13^ found the immune cell composition of tumor environment was associated with renal cell carcinoma patients’ progression‐free survival. Hirokazu Matsushita et al^14^ demonstrated that neoantigens together with β_2_M or HLA‐A expression were able to predict the clinical outcomes of patients with kidney cancer. However, these investigations mainly focus on clear cell renal carcinoma. The tumor immune environment for KIRP is still poorly understood. Besides, feasible immune biomarkers for the prediction of KIRP patients’ prognosis and possible new immune targets for KIRP treatment are very lacking. Therefore, it is essential to find a robust immune signature for KIRP which can serve as a predictor for KIRP patients’ survival from the perspective of tumor immunology and may become targets for immunotherapy of KIRP.

In this study, we utilized the transcriptome data from The Cancer Genome Atlas (TCGA) to develop and validate an immune‐related risk signature consisting of 15 immune‐related genes for KIRP. To evaluate the clinical value of the immune signature, we analyzed the correlation between the signature and clinical factors. And in order to further investigate the molecular and immune profiling of the signature, we conducted researches on the relationship of the immune signature with PBRM1 and SETD2 mutations, copy number variation (CNV), and immune‐related phenotypes.

## 2 METHODS

### 2.1 Patient samples and immune‐related genes

Clinical and transcriptomic data of KIRP samples were collected from UCSC‐TCGA (http://xena.ucsc.edu/), as shown in Table S1. The dataset was divided into training (n = 205) and testing set (n = 80). The transcriptome profiling of RNA expression was obtained by RNA‐seq and measured by fragments per kilobase of exon model per million mapped reads or FPKM values. Log2‐based transformation was used for the normalization of RNA expression profiles. In order to make sure of the detection reliability, genes with FPKM values being equal to 0 in more than 50% of the samples were removed from further analysis. The Immunology Database and Analysis Portal (ImmPort) comprehensive list of immune‐related genes were downloaded from the ImmPort database (https://immport.niaid.nih.gov),^15^ containing a total of 1534 immune‐related genes (Table S2). These immune‐related genes function as a variety of roles in immune pathways including antigen processing and presentation, B‐cell receptor signaling pathway, chemokine, chemokine receptors, cytokines, cytokines receptors, interferons, interferon receptors, interleukins, interleukin receptors, natural killer cell cytotoxicity, T‐cell receptor signaling pathway, transforming growth factor‐b (TGF‐b) family member, TGF‐b family member receptor, tumor necrosis factor (TNF) family members, and TNF family member receptors. These immune‐related genes were used to analyze the possible elected genes for constructing the immune‐related risk signature.

### 2.2 Construction of the immune‐related risk signature

All patients were randomly assigned to a training set (n = 205) (70% for identifying key immune‐related genes) and a testing set (n = 80) (30% for validating the immune‐related genes signature). Univariate analysis was used to identify immune‐related genes with prognostic ability (*P* < 0.05). The Cox proportional hazards model with an elastic‐net penalty (iteration = 1000) was performed using the R package called “glmnet” in order to identify the best gene model for predicting the prognosis in KIRP patients.^16^, ^17^ We estimated the penalty parameter by 10‐fold cross‐validation in training dataset. Genes weighted value was calculated based on a linear combination of Cox coefficient and gene expression.^19^, ^20^, ^21^

$$\text{Risk score}=\sum_{i=1}^{\text{N}}\left(\text{Expi*Coei}\right)$$

N, Expi, and Coei represented the number of signature genes, gene expression level, and coefficient value, respectively. The formula was used to calculate risk score, and the cutoff value of high and low risk was set as the median.

### 2.3 Performance assessment

To validate the prognostic capability of the immune‐related risk signature, we calculated the area under the curve (AUC) with R package “survivalROC” to evaluate the significance of the survival difference between high‐risk group and low‐risk group.^22^ Harrell's c‐index was utilized to indicate the predictive ability of the risk signature in training, testing, and the total cohort. The Kaplan‐Meier (K‐M) survival curves together with the Cox proportional hazards model were performed using the R package called “survival” (https://CRAN.R-project.org/package=survival) 23 and “BhGLM” (https://www.soph. uab.edu/ssg/software/bhglm).^24^ The multivariate analysis was performed to assess the independent prognostic ability of the immune‐related risk signature.

### 2.4 Mutation analysis

The mutation data available for 281 TCGA KIRP patients with mutation information were downloaded from Genomic Data Common (GDC) (https://portal.gdc.cancer.gov/). The analysis data containing somatic variants were stored in the form of Mutation Annotation Format (MAF).We calculated each gene mutation rate of patients in high risk or low risk as follows:

$$\text{Gene mutation rate =}\;\frac{\text{Gi}}{\text{Pi}}$$

Gi represented the number of patients with the gene mutation in each group; Pi represented the total number of patients. Survival analysis was performed in top 20 significantly mutation genes with R packages maftools.^25^ Gene mutation profiles were also shown with maftools.

### 2.5 CNV analysis

We downloaded whole genome microarray contained gene‐level copy number variation (GISTIC‐preanalyzed data) and SNP array data contained Masked Copy Number Segment. The metadata information of UCSC‐TCGA database shown that GISTIC2 further thresholded the estimated values to −2, −1, 0, 1, 2, representing homozygous deletion, single copy deletion, diploid normal copy, low‐level copy number amplification, or high‐level copy number amplification. Therefore, we considered amplifications (GISTIC value 1 and 2) and deletions (GISTIC value −1 and −2) together as a general index for CNVs represented. *t* test was performed between CNV and no‐CNV genes to assess differential risk score (*P* < 0.05). CNV rates of high risk and low risk were calculated based on this reference.^26^ R package “copynumber” is available for visualization of the segmentation results.^27^, ^28^

### 2.6 Gene set enrichment analysis

To analyze the immune‐related gene ontology (GO) terms of the immune‐related risk signature, gene set enrichment analysis (GSEA) was performed between high‐risk and low‐risk phenotypes (https://pypi.org/project/gseapy/).^29^ Gene ontology gene sets were downloaded from Molecular Signatures Database (MSigDB) (http://software.broadinstitute.org/gsea/downloads.jsp). We considered the enriched gene sets to be statistically significant in GSEA when the nominal *P* value was less than 0.05, and the false discovery rate (FDR) was less than 0.25.

### 2.7 Statistical analysis

The heatmaps were generated by applying R package “ComplexHeatmap” R package.^30^ The boxplots were conducted using the R package called “ggplot2”.^31^ We calculated c‐index with R package “survcomp”.^32^ The Student's *t* test was used for statistical comparison of paired data. The ANOVA test was conducted for comparison of more than two scores. Pearson's chi‐square tests were performed for comparison of categorical variables. Exact test was performed using R package “stats” version 3.5.1. The statistical analysis of this research was conducted by R language (https://www.r-project.org/). A *P* value <0.05 was thought to be statistically significant.

## 3 RESULTS

### 3.1 Construction and validation of the immune‐related risk signature

**Figure 1:**
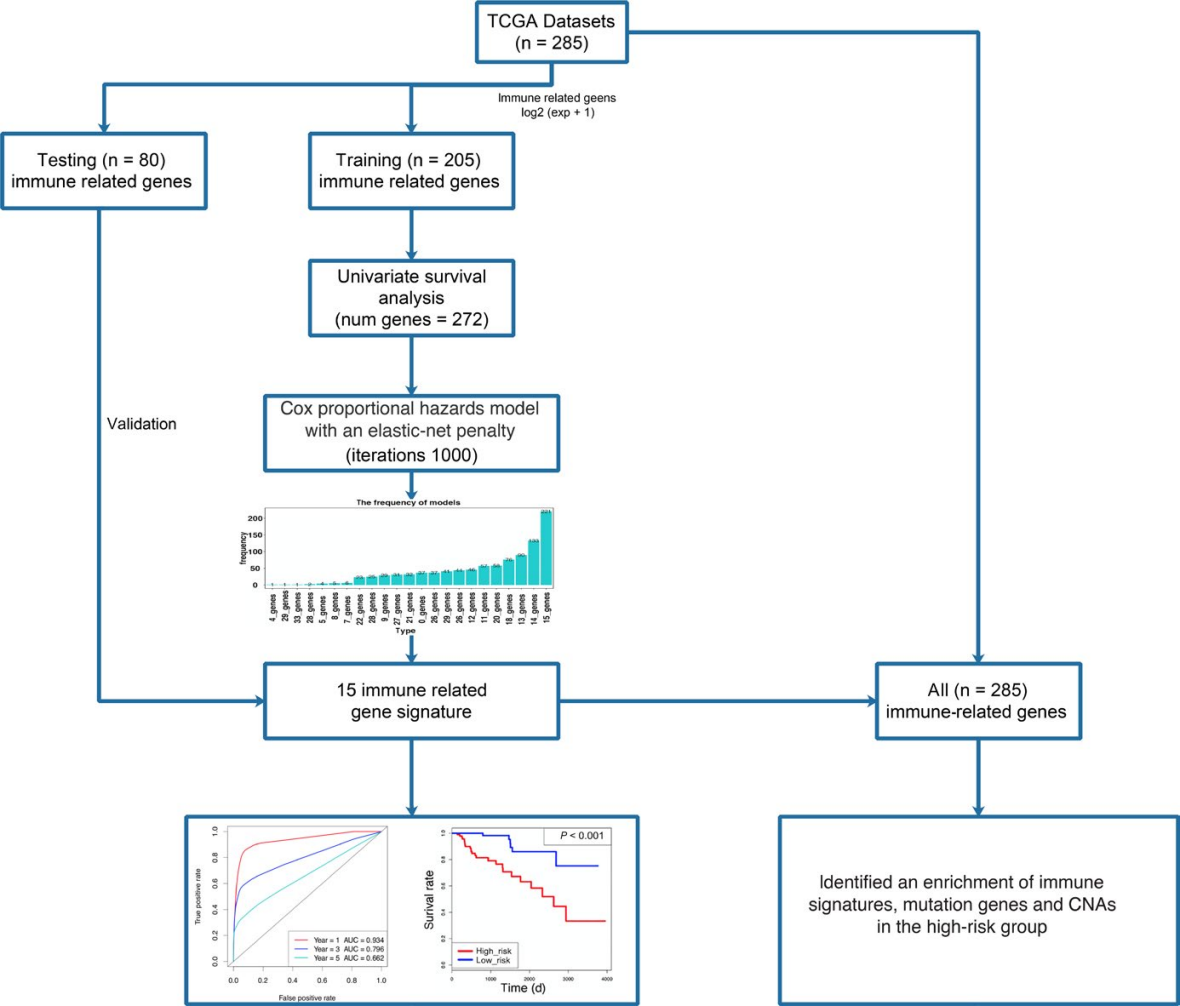
The workflow describing the schematic overview of the project

**Figure 2:**
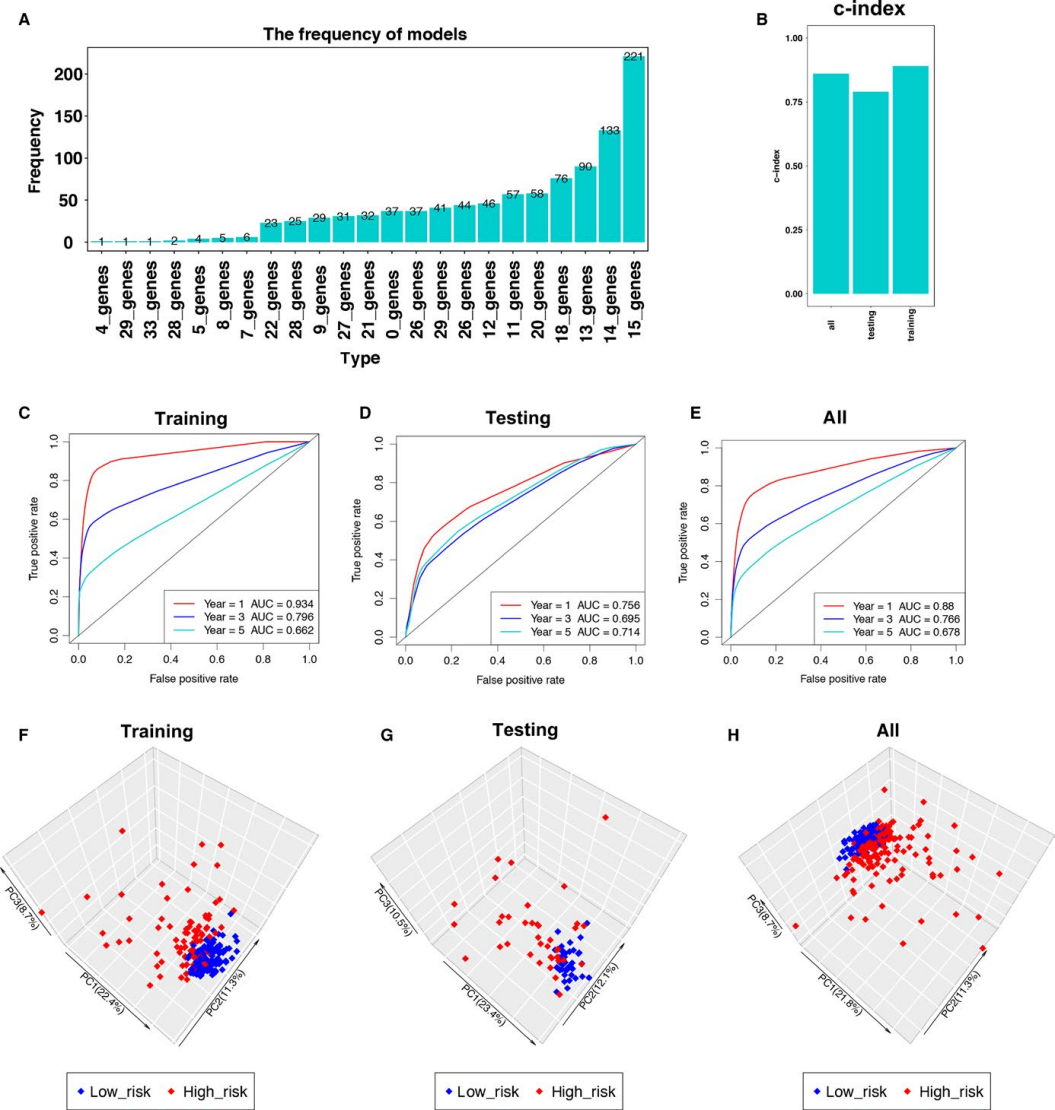
Construction and validation of the immune‐related risk signature. A, Results of elastic net. After 1000 iterations, there were 23 gene groups, of which 15 immune‐related genes were significantly higher frequency than other gene groups. B, The c‐indexes for training, testing, and total cohort were 0.891, 0.790, and 0.861, respectively. C‐E, Time‐dependent ROC curve analysis of the signature in training set, testing set, and all set. 1‐year AUC, 3‐year AUC, and 5‐year AUC in training set, testing set, and all set is 0.934, 0.756, 0.88, 0.796, 0.695, 0.766, 0.662, 0.714, and 0.678, respectively. F‐H, Principal component analysis of the training, testing, and total KIRP cohort with the 15‐immune‐related gene expression. The high‐risk patients were marked by red dots, and the low‐risk patients were marked by blue dots

**Figure 3:**
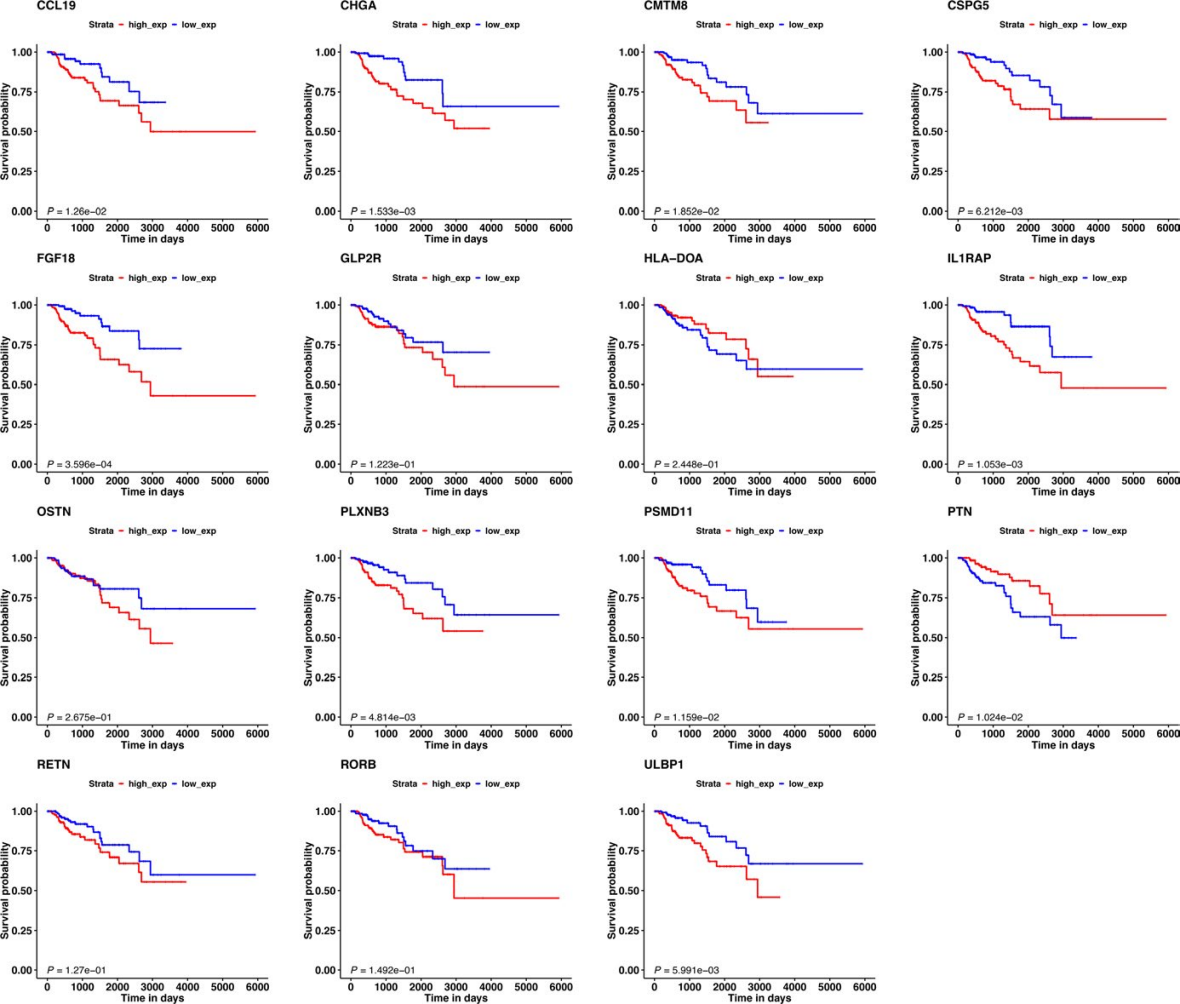
The K‐M analysis of the 15 immune‐related genes used to construct the immune‐related risk signature for KIRP, including CCL19, CHGA, CMTM8, CSPG5, FGF18, GLP2R, HLA‐DOA, IL1RAP, OSTN, PLXNB3, PSMD11, PTN, RETN, RORB, and ULBP1. The expression of CCL19, CHGA, CMTM8, CSPG5, FGF18, IL1RAP, PLXNB3, PSMD11, and ULBP1 was positively related with KIRP patients’ prognosis. The expression of PTN was negatively with KIRP patients’ prognosis. The expression of GLP2R, HLA‐DOA, OSTN, RETN, and RORB showed no significantly correlated with KIRP patients’ prognosis

The workflow of our study is illustrated in Figure 1. The training set was used for construction of the immune‐related risk signature. The testing set was used for validation. Using univariate analysis, we identified 272 genes with predicting prognosis ability from a total of 1534 immune‐related genes. Then, the 272 genes underwent the elastic net to construct an immune‐related risk signature. After 1000 iterations, there were 23 gene groups, of which 15 immune‐related genes were elected to form an immune‐related risk signature. The characteristics of the 23 gene groups were shown in Table S3. The 15 immune‐related genes were chosen because of its significantly higher frequency than other gene groups, as shown in Figure 2A. This 15‐gene model achieved the frequency of 221 times, which accounted for more than 20% in 1000 iterations. The univariate analysis of the 15 genes is demonstrated in Table 1, and the K‐M analysis of the genes is demonstrated in Figure 3. Risk score was estimated as follows:

**Table 1:** Univariate Cox analysis for overall survival of 15 immune‐related genes in training set

Gene name	HR	95% CI	*P* value
HLA‐DOA	0.552	0.309‐0.985	0.044
PSMD11	439.498	27.710‐6970.830	<0.001
ULBP1	7.636	3.389‐17.205	<0.001
CCL19	2.208	1.591‐3.065	<0.001
PLXNB3	3.136	2.000‐4.919	<0.001
CHGA	2.338	1.594‐3.429	<0.001
CMTM8	21.090	6.769‐65.709	<0.001
CSPG5	14.031	6.296‐31.269	<0.001
FGF18	11.878	4.671‐30.200	<0.001
OSTN	4.081	1.032‐16.134	0.045
PTN	0.645	0.471‐0.883	0.006
RETN	1.971	1.137‐3.418	0.016
GLP2R	4.584	2.095‐10.033	<0.001
IL1RAP	6.319	3.167‐12.606	<0.001
RORB	3.480	1.191‐10.171	0.023

CI, confidence interval; HR, hazard ratio.

Risk score = (−0.006*HLA‐DOA) + (0.634*PSMD11) + (0.388*ULBP1) + (0.143*CCL19) + (0.154*PLXNB3) + (0.341*CHGA) + (0.208*CMTM8) + (0.350*CSPG5) + (0.355*FGF18) + (0.330*OSTN) + (−0.003*PTN) + (0.137*RETN) + (0.398*GLP2R) + (0.593*IL1RAP) + (0.928*RORB).

Then, the c‐index for the training, testing, and total set was 0.891, 0.790, and 0.861, respectively (Figure 2B, *P* < 0.0001). The ROC curve analysis of the signature in training set demonstrated the promising predictive value of it for KIRP‐specific survival (1‐year AUC = 0.934, 3‐year AUC = 0.796, 5‐year AUC = 0.662, Figure 2C). After that, we validated the signature in the testing set. In the testing set, the 1‐year AUC was 0.756, 3‐year AUC was 0.695, and 5‐year AUC was 0.714 (Figure 2D). As for the total cohort, the 1‐year AUC was 0.88, 3‐year AUC was 0.766, and 5‐year AUC was 0.678 (Figure 2E). Principal component analysis of the training, testing, and total KIRP cohort demonstrated a different distribution pattern of high risk and low risk based on 15 immune‐related gene expression, indicating their difference in immune phenotype (Figure 2F‐G).

### 3.2 Correlation of the immune‐related risk signature with clinicopathologic features

**Figure 4:**
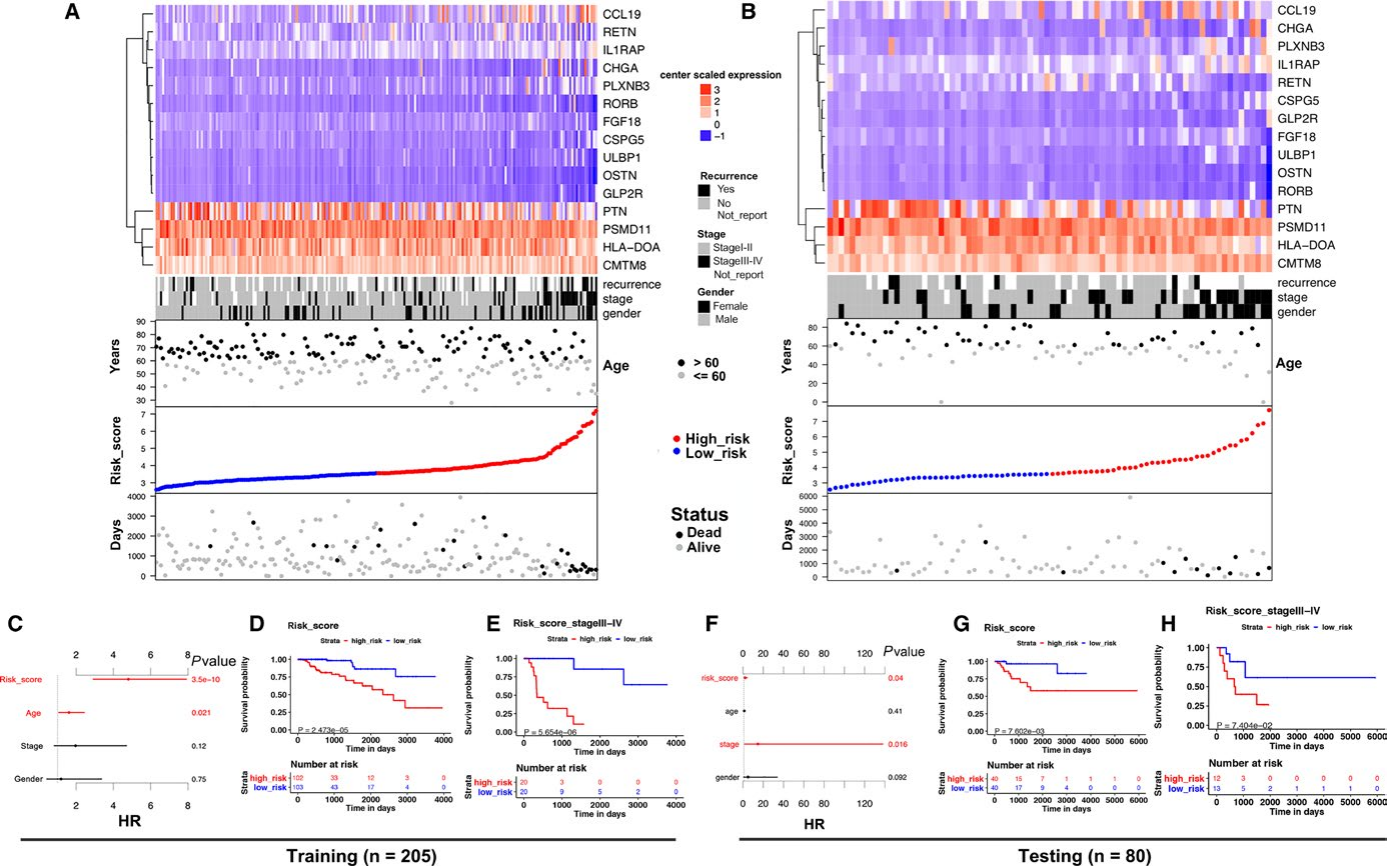
15 immune‐related gene predictor‐score analysis of KIRP patients in both the training and testing cohorts. A and B, Heatmap showed the 15‐immune‐related gene expression distribution in training, testing cohorts. Each column represented the same patient corresponded to the below point showing risk score distribution, survival status, and time in KIRP patients. Each point represented one patient sorted by the rank of the risk score. Red, blue, black, and gray represented patient high risk, low risk, dead, and alive, respectively. The patients in female, advanced stage, and tumor recurrence showed high‐risk score. C and F, The multivariate Cox analysis in training and testing cohorts. The 15 immune‐related genes signature was able to serve as an independent prognostic factor for OS. D and G, The survival analysis in training and testing cohorts without stratification. Survival curve showed that patients with high‐risk score were correlated with a trend toward worse survival outcomes. E and H, The survival analysis in stage III‐IV patients in training and testing cohorts. Survival curve showed that patients with high‐risk score were correlated with a trend toward worse survival outcomes

**Figure 5:**
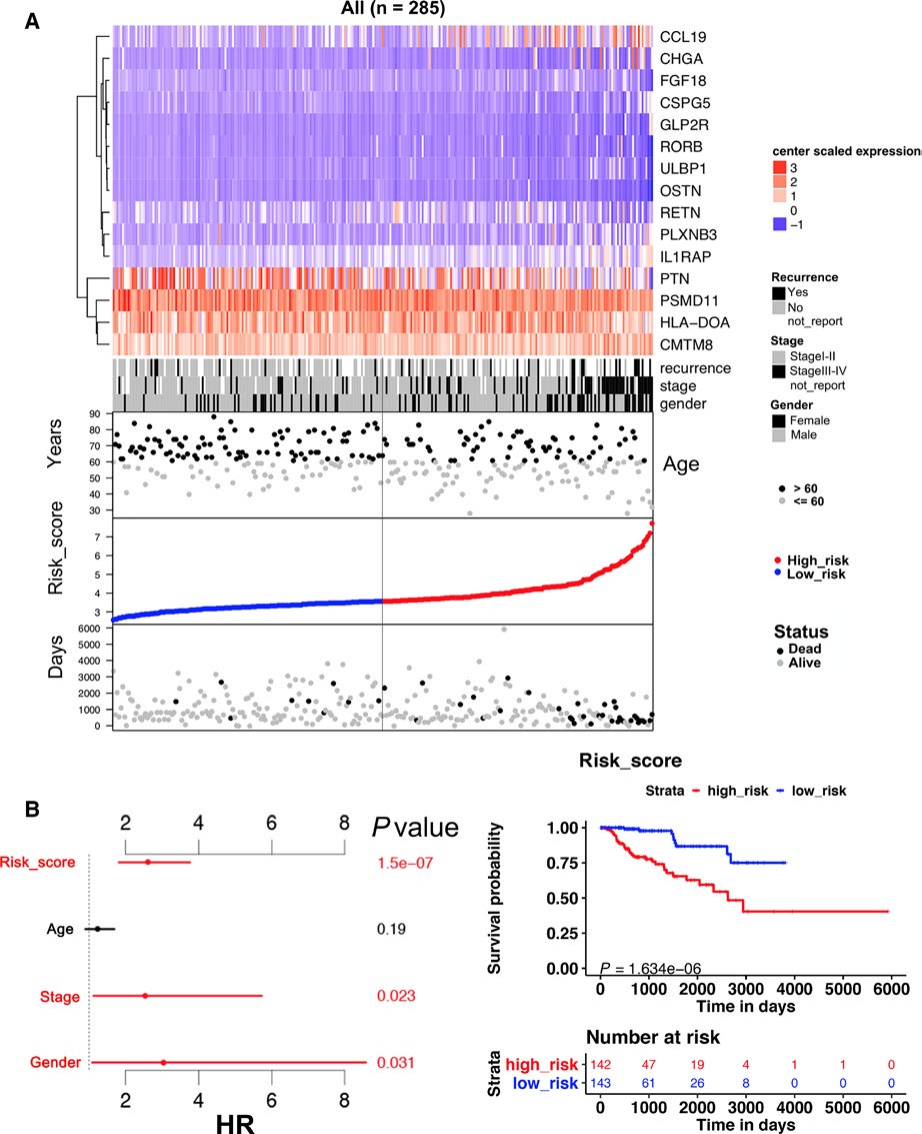
15 immune‐related gene predictor‐score analysis of KIRP patients in all cohort. A, Heatmap showed the 15 immune‐related gene expression distribution in all cohort. Each column represented the same patient corresponded to the below point showing risk score distribution, survival status, and time in KIRP patients. Each point represented one patient sorted by the rank of the risk score. Red, blue, black, and gray represent patient high risk, low risk, dead, and alive, respectively. The patients in female, advanced stage, and tumor recurrence showed high‐risk score. B, (Left)The multivariate Cox analysis in all cohort. The 15 immune‐related genes signature was able to serve as an independent prognostic factor for OS (*P* < 0.001). (Right) The survival analysis in all cohort without stratification. Survival curve showed that patients with high‐risk score were correlated with a trend toward worse survival outcomes (*P* < 0.001)

**Figure 6:**
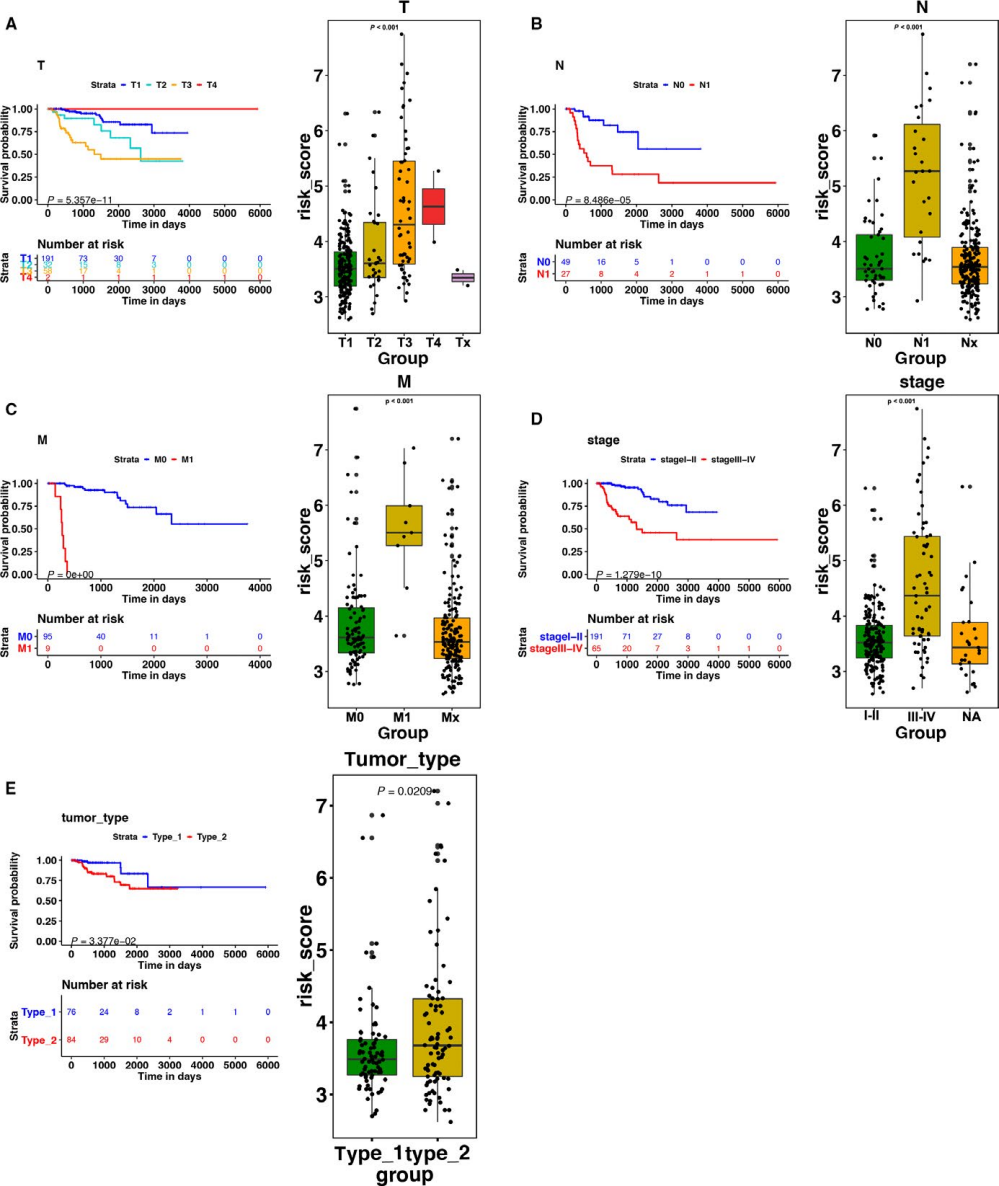
(Left)The K‐M analysis of (A) T stage, (B) N stage, (C) M stage, and (D) staging. (right) The boxplots of the relationship between the signature and (A) T stage, (B) N stage, (C) M stage, (D) staging, and (E) tumor type. Patients with higher level of T, N, M stages, advanced staging, and type 2 KIRP tended to have higher risk score

The 15 immune‐related genes formed the signature exhibited distinct expression diverse expression patterns, including four relatively high‐expression genes (PTN, PSMD11, HLA‐DOA, and CMTM8) and 11 relatively low‐expression genes (CCL19, RETN, IL1RAP, CHGA, PLXNB3, RORB, FGF18, CSPG5, ULBP1, OSTN, and GLP2R), as shown in Figure 4A,B, and 5A. Afterward, we assessed whether there was statistically different in the distribution of clinicopathologic factors between low‐risk and high‐risk groups. The heatmap demonstrated that the high‐risk group was correlated with female, advanced stage, and tumor recurrence in training, testing, and total cohorts (Figure 4A,B and 5A). The relationship between the signature and staging and tumor type further found that patients with higher level of T, N, M stages, and type 2 KIRP tended to have higher risk score. (*P* < 0.05, Figure 6A‐E, Table S4).

### 3.3 Association between the immune‐related risk signature and patients' survival outcomes

**Figure 7:**
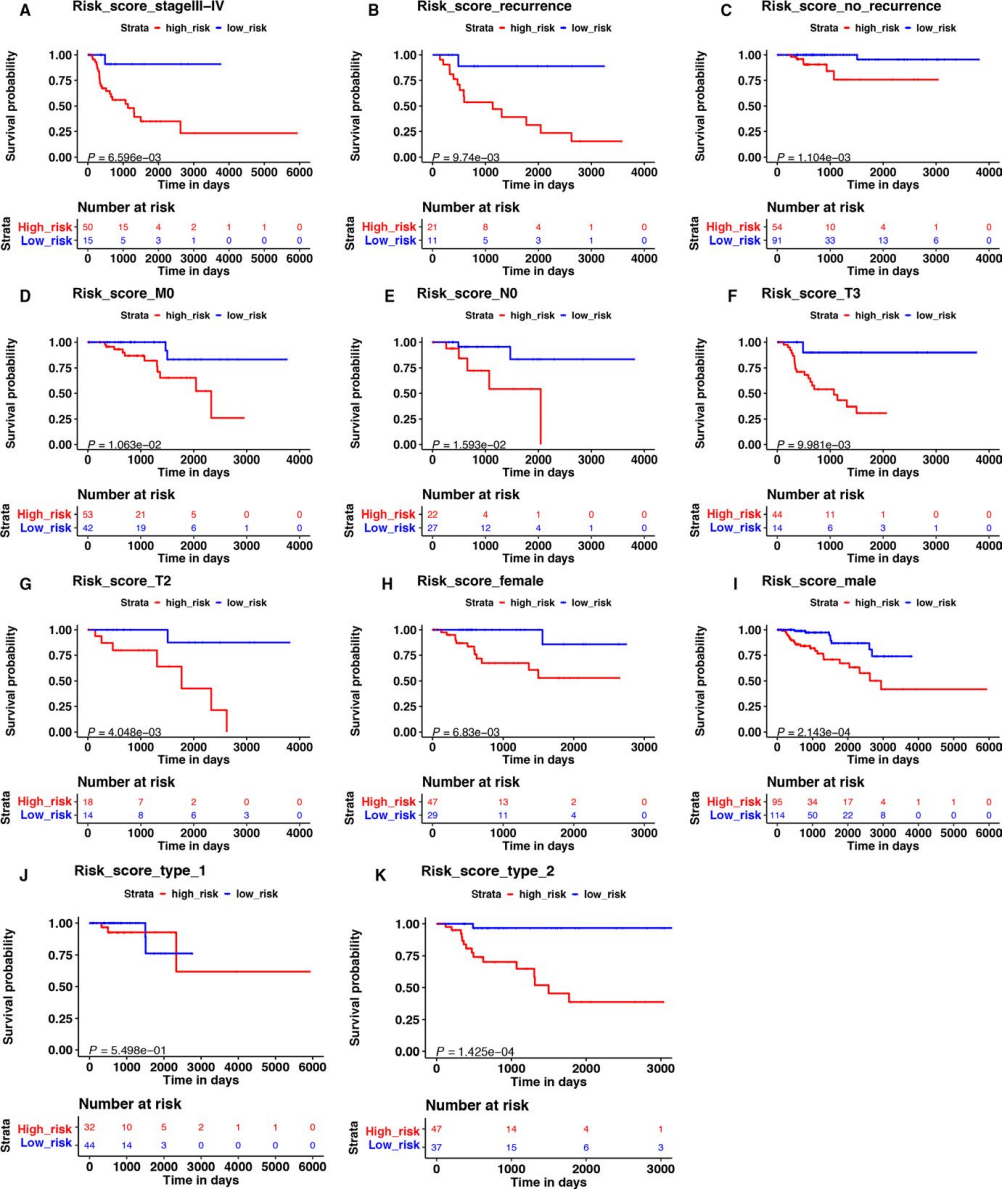
The K‐M analysis of the risk signature grouping according to patients with (A) stage III‐IV, (B) recurrence, (C) no recurrence, (D) M0 stage, (E) N0 stage, (F) T3 stage, (G) T2 stage, (H) female, (I) male, (J) type 1 KIRP, and (K) type 2 KIRP

The K‐M analysis demonstrated that patients with high‐risk score were correlated with a trend toward worse survival outcomes in training (*P* < 0.001, Figure 4D), testing, (*P* < 0.01, Figure 4G), and total sets (*P* < 0.001, Figure 5B). Then, we further conducted the K‐M analysis for stage III‐IV KIRP patients, considering the positive correlation between the immune‐related risk signature and tumor stage. We found that the overall survival (OS) of advanced stage patients was also positively correlated with high‐risk score in all of the three cohorts (*P* < 0.05, Figure 4E,H, Figure 7A). We also demonstrated that the risk signature could predict the OS of subgroup of KIRP, including patients with recurrence (Figure 7B, *P* < 0.001), no recurrence (Figure 7C, *P* < 0.001), M0 stage (Figure 7D, *P* < 0.001), N0 stage (Figure 7E, *P* < 0.001), T3 stage (Figure 7F, *P* < 0.001), T2 stage (Figure 7G, *P* < 0.001), female (Figure 7H, *P* < 0.001), male (Figure 7I, *P* < 0.001), and type 2 KIRP (Figure 7K, *P* < 0.001). However, there was no correlation between the risk score and patients’ OS in type 1 KIRP patients (Figure 7J). The univariate Cox analysis of risk signature and clinical parameters in training, testing, and total group is demonstrated in Table 2. In the multivariate analysis, the signature was able to serve as an independent prognostic factor for OS with a HR of 4.800 in training group (95% confidence interval [95%CI] = 2.941‐7.836, *P* < 0.001, Figure 4C, Table 3), 2.079 in testing group (95%CI = 1.035‐4.176, *P* < 0.05, Figure 4F, Table 3), and 2.613 in the total cohort (1.826‐3.738, *P* < 0.001, Figure 5B, Table 3). But we found that the signature just exhibited prognostic ability in training and total group after we included the T, N, and M stages as independent clinical parameters (Tables S5 and S6). This might be caused by the small sample size since we excluded cases with unknown T, N, or M stages. Clinicopathologic features (age, stage, and gender) failed to exhibit a constantly independent role for predicting KIRP survival outcomes in all of the three sets.

**Table 2:** Univariate Cox analysis for overall survival of risk signature and clinical parameters in training, testing, and all groups

	Variable	HR	95% CI	*P* value
Training	Risk score (low risk vs high risk)	5.824	3.708‐9.147	<0.001
	Age (≤60 vs >60)	1.012	0.977‐1.049	0.4901
	Stage (I and II vs III and IV)	4.837	2.292‐10.209	<0.001
	Gender (male vs female)	0.459	0.196‐1.075	0.073
Testing	Risk score (low risk vs high risk)	1.930	1.334‐2.793	<0.001
	Age (≤60 vs >60)	0.995	0.965‐1.027	0.771
	Stage (I and II vs III and IV)	22.461	2.873‐175.59	0.003
	Gender (male vs female)	0.818	0.238‐2.817	0.750
All	Risk score (low risk vs high risk)	2.613	2.100‐3.252	<0.001
	Age (≤60 vs >60)	0.996	0.969‐1.025	0.794
	Stage (I and II vs III and IV)	6.473	3.362‐12.462	<0.001
	Gender (male vs female)	0.591	0.297‐1.178	0.135

CI, confidence interval; HR, hazard ratio.

**Table 3:** Multiple Cox analysis for overall survival of risk signature and clinical parameters in training, testing, and all groups

	Variable	HR	95% CI	*P* value
Training	Risk score (low risk vs high risk)	4.800	2.941‐7.836	<0.001
	Age (≤60 vs >60)	1.610	1.073‐2.416	0.021
	Stage (I and II vs III and IV)	1.967	0.837‐4.618	0.121
	Gender (male vs female)	1.183	0.426‐3.288	0.747
Testing	Risk score (low risk vs high risk)	2.079	1.035‐4.176	0.040
	Age (≤60 vs >60)	1.245	0.737‐2.103	0.413
	Stage (I and II vs III and IV)	14.686	1.635‐131.906	0.016
	Gender (male vs female)	5.003	0.771‐32.46	0.092
All	Risk score (low risk vs high risk)	2.613	1.826‐3.738	<0.001
	Age (≤60 vs >60)	1.230	0.902‐1.679	0.191
	Stage (I and II vs III and IV)	2.531	1.135‐5.641	0.023
	Gender (male vs female)	3.048	1.105‐8.408	0.031

CI, confidence interval; HR, hazard ratio.

### 3.4 Involvement of mutations in the immune‐related risk signature

**Figure 8:**
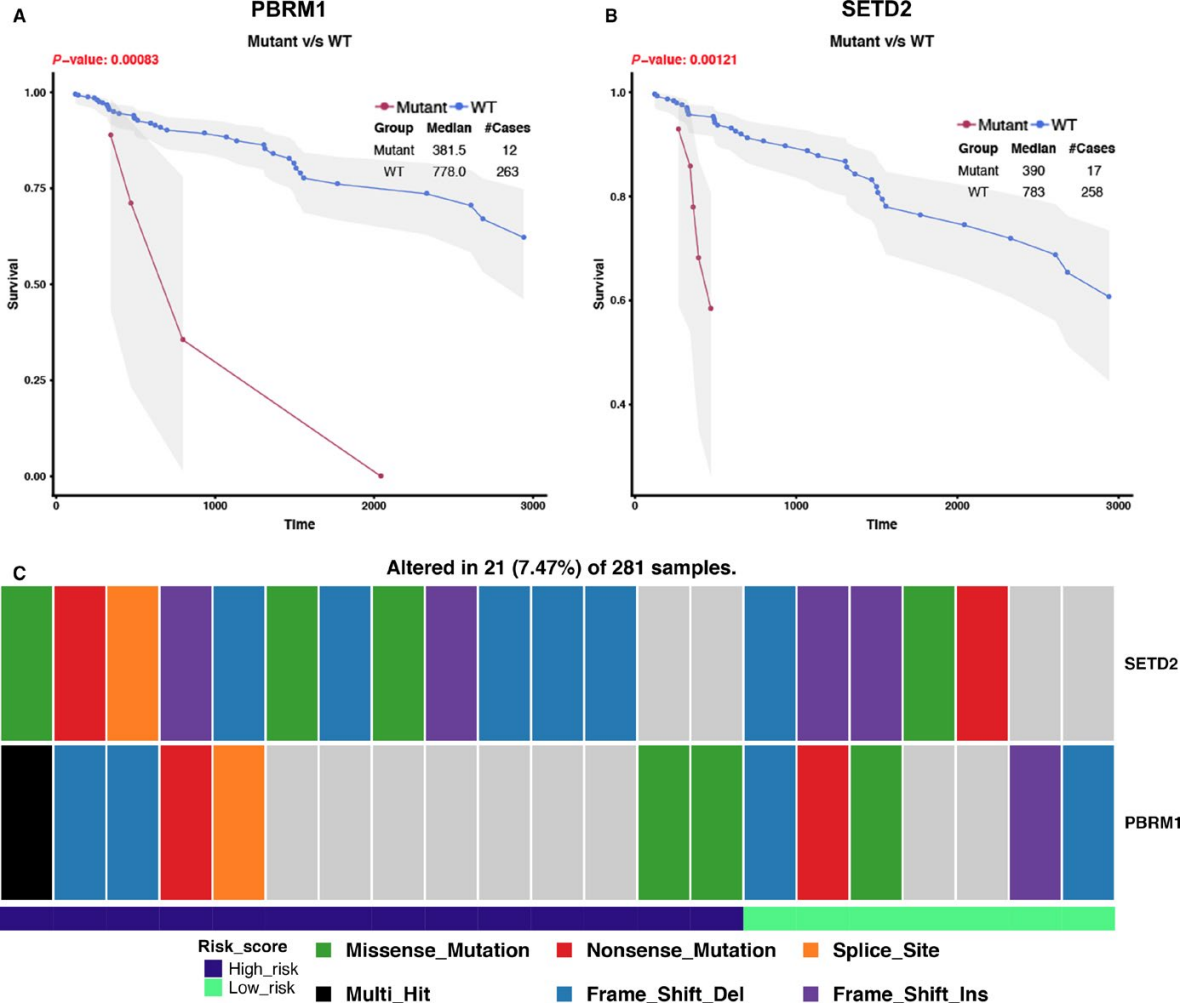
Significant mutation genes with prognostic value in KIRC in 281 TCGA patients with mutation information (137 high‐risk patients and 138 low‐risk patients and six patients without expression data). A and B, The mutant status of PBRM1 and SETD2 was closely related with patients' prognosis. C, The mutation profiles of PBRM1 and SETD2 between high‐ and low‐risk groups. High‐risk group achieved higher mutation frequency than low‐risk group (SETD2: high risk 4.27% vs low risk 1.78%; PBRM1: high risk 2.49% vs low risk 1.78%)

In order to investigate whether the mutations of genes were associated with the immune‐related risk signature, we first identified mutations with prognostic ability in KIRP. The mutational analysis was conducted on the 20 highest frequently mutated genes in 281 TCGA patients with mutation information (Figure S1)). Among the 20 highest frequently mutated genes, PBRM1 and SETD2 mutations were strongly associated with patients’ poor survival outcomes compared the wide type (Figure 8A,B). For PBRM1, the mutation rate for high‐risk patients was 2.49%, while 1.78% for low‐risk patients (*P* = 0.361). For SETD2, the mutation rate for high‐risk patients was 4.27%, while 1.78% for low‐risk patients (*P* < 0.01). The mutation profiles of the two genes between high‐ and low‐risk groups were presented in Figure 8C, which indicated that high‐risk group achieved higher mutation frequency than low‐risk group.

### 3.5 Correlation of the immune‐related risk signature with copy number variations (CNV)

**Figure 9:**
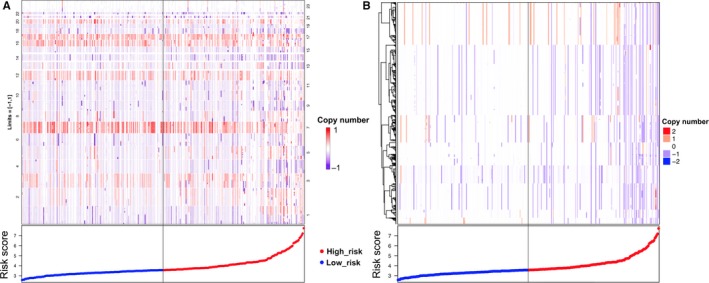
Correlation of the immune‐related risk signature with copy number variations. A, Chromosome location and segment mean data are presented. The clustering of somatic copy number alterations showed that the high‐risk patients had more significant chromosome aberrations. B, Gene‐level copy number variation. Clustering of gene somatic copy number alterations showed significant chromosome deletion aberrations in high‐risk group

To elucidate whether there was a relationship between the signature and CNV, we analyzed the CNV data in the TCGA. In high‐risk group, clustering of somatic copy number alterations showed the more significant chromosome aberrations than low risk (Figure 9A). High‐risk group had higher CNV rate than low‐risk group (high risk vs low risk：4305/141 vs 10545/142, *P* < 0.001). *t* test was performed between CNV and no‐CNV genes to assess differential risk score. We identified 2957 genes with differential risk score between CNV and no‐CNV (*P* < 0.05). Clustering of these gene somatic copy number alterations showed significant chromosome deletion aberrations in high‐risk group (Figure 9B).

### 3.6 Involvement of immune‐related gene ontology terms by the immune‐related risk signature

**Figure 10:**
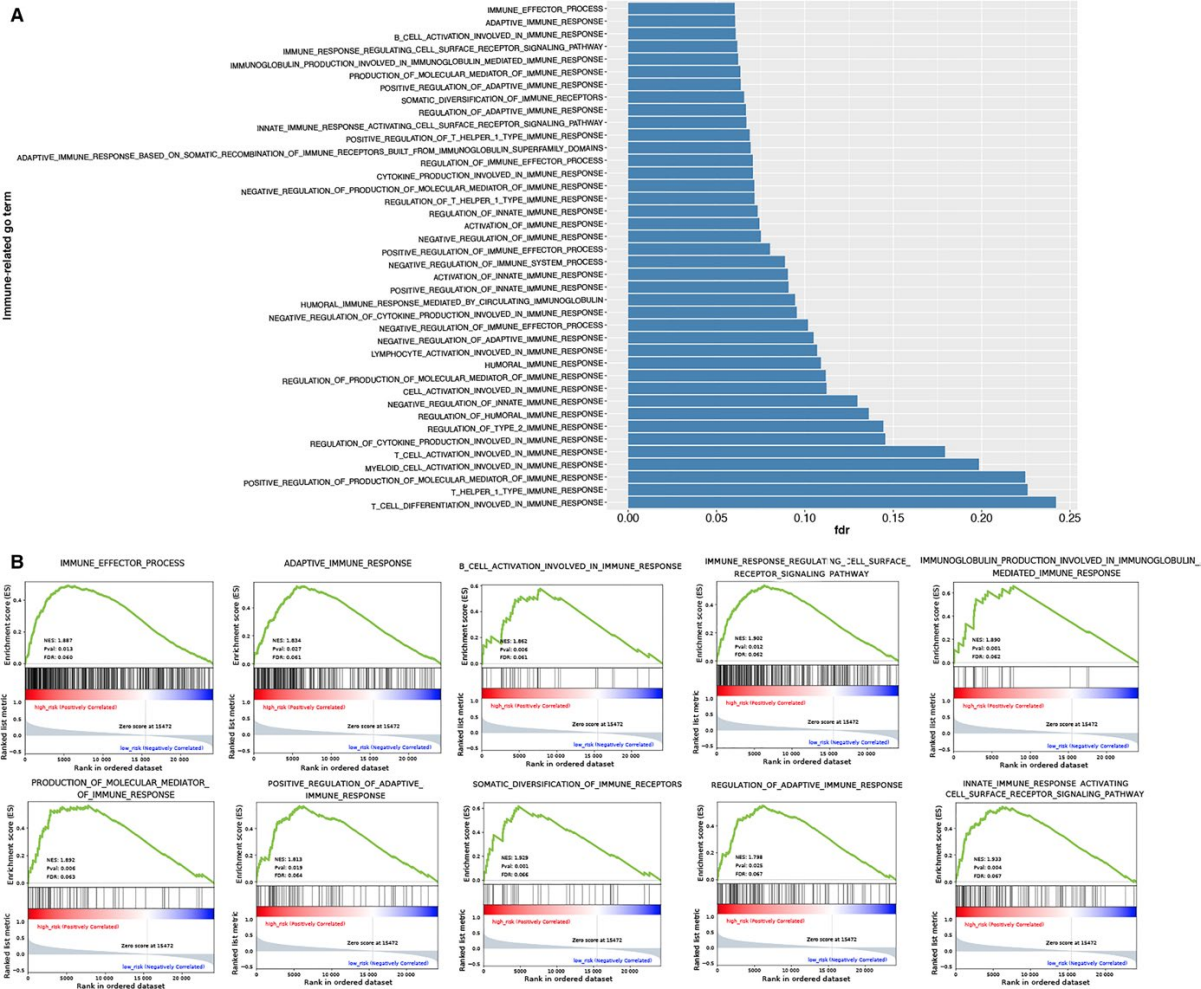
Gene set enrichment analysis for comparing immune phenotype between high‐risk group and low‐risk group. A, 40 significant immune‐related GO terms enrichment between high‐risk group and low‐risk group with FDR <0.25. B, Significant enrichment of 10 immune‐related GO terms in high‐risk group

The GSEA was performed for functional annotation of the immune‐related risk signature. The results demonstrated that there was a total of 40 immune‐related gene ontology terms with FDR <0.25 (Figure 10A, Table S7). We further demonstrated the GSEA results of the top 10 immune‐related gene ontology terms in Figure 10B, including immune effector process, adaptive immune response, B‐cell activation involved in immune response, immune response regulating cell surface receptor signaling pathway, immunoglobulin production involved in immunoglobulin‐mediated immune response, production of molecular mediated immune response, positive regulation of adaptive immune response, somatic diversification of immune receptors, regulation of adaptive immune response, and innate immune response activating cell surface receptor signaling pathway. The GSEA results demonstrated that genes within the 10 immune‐related pathways were shown to enrich in the group with high‐risk. Therefore, we suggested that the immune‐related risk signature demonstrated an intensive immune phenotype. In order to further investigate more immune‐related mechanism of the signature, we evaluated the relationship between the risk score and the expression of T‐cell markers including CD4 and CD8A and immune checkpoints including PD‐1 and cytotoxic T‐lymphocyte associated protein 4 (CTLA‐4). We found that patients in high‐risk group tended to have more CD8+ (*P* < 0.001, Figure S2A) and CD4+ T‐cell infiltration (*P* < 0.001, Figure S2B). Besides, high‐risk group patients expressed higher level of programmed cell death ligand 2 (PD‐L2) (*P* < 0.001, Figure S2C), PD‐1 (*P* < 0.05, Figure S2D), and CTLA‐4 (*P* < 0.01, Figure S2E). The expression of PD‐L1 was also higher in high‐risk group patients than those in low‐risk group, while there was no statistical significance (Figure S2F).

## 4 DISCUSSION

The KIRP is always less studied than clear cell type in renal cancer, considering its low incidence. This leads to the difficulty of in‐depth investigation for the treatment strategies and prognostic prediction of KIRP. Taking into account the importance of immune environment in the progression of cancer,^33^ it is essential to find out immune‐related biomarker for the prognosis of KIRP patients, which may also serve as a significant role in immunotherapy. Our study established a robust immune‐related risk signature for KIRP using the TCGA KIRP datasets that was able to predict the patients’ survival outcomes and was significantly correlated with clinicopathologic features. More importantly, we found this signature was associated with CNV and was involved in many immune‐related gene ontology terms. These findings suggest the value of our signature for KIRP patients’ prognosis and possible immune targets for immunotherapy.

The immune‐related signature consisted of 15 immune‐related genes with prognostic ability. In our signature, we found 11 of the 15 genes were cytokines or cytokine receptors, functioning as significant parts in inflammatory process of tumor initiation and progression.^34^, ^35^ This can be further understood by the explanation that cytokines and their receptors can activate potential oncogenic transcription factors in STAT and NFκB families to promote the pathogenesis of cancer.^36^ Therefore, KIRP patients with high‐immune risk of our established signature can reflect to an increased tumor inflammatory microenvironment, which facilitates the progression of KIRP and leads to the poor OS of patients. More interestingly, in the 15 immune‐related genes that consist of the signature, PSMD11 was the most significant factor in the univariate analysis (HR = 439.498). The PSMD11 protein consists of part of 26S proteasome which is involved in protein homeostasis through removing misfolded proteins.^37^ Hence, PSMD11 participates in the antigen processing process. Besides, PSMD11 was one of the suggested mechanisms for several neurodegenerative disorders such as Alzheimer's and Parkinson's disease.^38^ Therefore, we suggested the intriguing intersection of neuroscience and immuno‐oncology bridging by PSMD11. PTN was another gene in the signature that attracts our attention. There have been studies showing that PTN can promote the tumor microenvironment remodeling and transdifferentiation of macrophages.^39^, ^40^ This can further highlight the importance of our immune signature in the KIRP microenvironment.

In order to figure out the clinical values of this signature, the associations between the signature and clinicopathological factors and patients’ OS were evaluated. Patients with high‐immune risk score tended to be female, advanced stage, tumor recurrence, and type II KIRP. Our investigation was the first to find the immune‐related differences between the different clinical cohorts in KIRP. It can also be referred that the differences in tumor immunity may reflect the OS of different clinical cohorts. Patients with high‐immune risk may own a tumor immune microenvironment that can promote the development and recurrence of KIRP, which leads to advanced stage and relapse of tumor. Therefore, our signature can not only predict patients’ survival outcomes but predict the possibility of disease progression and relapse. The multivariate analysis can further confirm the immune‐related risk signature to be an independent predictor for clinical KIRP patients. Thereby, combining this signature with other clinical factors could serve as a promising tool for the prognosis of KIRP patients in the future.

Furthermore, we tried to investigate the molecular mechanisms of the immune‐related risk signature. Our results demonstrated that high‐risk patients tended to have PBRM1 and SETD2 mutations than those of low‐risk, which also indicates that the prognostic ability of our signature. Interestingly, our study found there was a positive relationship between the risk signature and chromosome deletion aberrations. Chromosome aberrations contain many immune system genes, such as chemokine (CXC‐motif) ligand 1 (CXCL1), CXCL10, cytokine‐dependent hematopoietic cell linker (CLNK), and alpha‐protein kinase 1 (ALPK1) (https://www.uniprot.org/docs/humchr04. txt).^17^ Hence, we consider that there may exist a connection between the 15 immune‐related genes of the signature and the immune system genes in chromosome deletion aberrations. The copy number loss of these immune system genes in chromosome may affect the expression or function of the 15 immune‐related genes and patients’ survival outcomes. Further studies are needed to verify our hypothesis. Finally, our GSEA can further prove the robust connection of the signature with immune systems. Patients with high‐risk score were more associated with immune‐related pathways, especially the adaptive immune response. Besides, high‐risk group patients had more CD8+ and CD4+ T‐cell infiltration, which reflect an immunological microenvironment of KIRP. Further study showed that high‐risk group patients tended to have higher PD‐L2, PD‐L1, PD‐1, and CTL‐4 expression in tumor environment. This indicates that despite the high infiltration of T cells in KIRP microenvironment, the function of T cells is inhibited by PD‐1‐ or CTL‐4‐mediated suppression pathways. Therefore, high‐risk patients were more likely to benefit from immune checkpoint blockade targeting PD‐1 and CTLA4. This also accords with the study of Yu‐Pei Chen et al^41^ their study also suggest that tumors with pre‐existing intratumor T cells that express high level of PD‐L1 and are suppressed by PD‐1/PD‐L1 pathway are most likely to benefit from immune checkpoint blockade. Nevertheless, further investigations are needed to evaluate the relationship between the signature and immunotherapy.

Taken together, our study was the first to identify and validate a 15 immune‐related gene‐based risk signature with the ability of being an independent prognosis predictor for KIRP patients. This could indicate the immune response intensity in the KIRP microenvironment, as suggested by the GSEA results. Our signature can also provide novel clinical applications for KIRP considering immune targets and immune‐related treatment. Our investigations have the advantages of using the massive cohort from TCGA database to find and validate the signature and the robust method of developing the immune‐related risk score. Nevertheless, our research has limitations of being a retrospective study with limited sample size, which may lead to the problem of overfitting. Therefore, a cohort with more patients is needed to solve this. Besides, our immune‐related signature should be applied to clinical environment to test its predictive ability, and the 15 immune‐related genes also need further functional analysis for their possible clinical usage.

## CONFLICT OF INTEREST

None declared.
