# Supplementary figures and images for: Construction of immune‐related risk signature for renal papillary cell carcinoma
Source: Cancer Med. 2018 Dec 5;8(1):289–304. doi: 10.1002/cam4.1905 (PMC6346237; doi:10.1002/cam4.1905)

Altered in 169 (60.14%) of 281 samples.

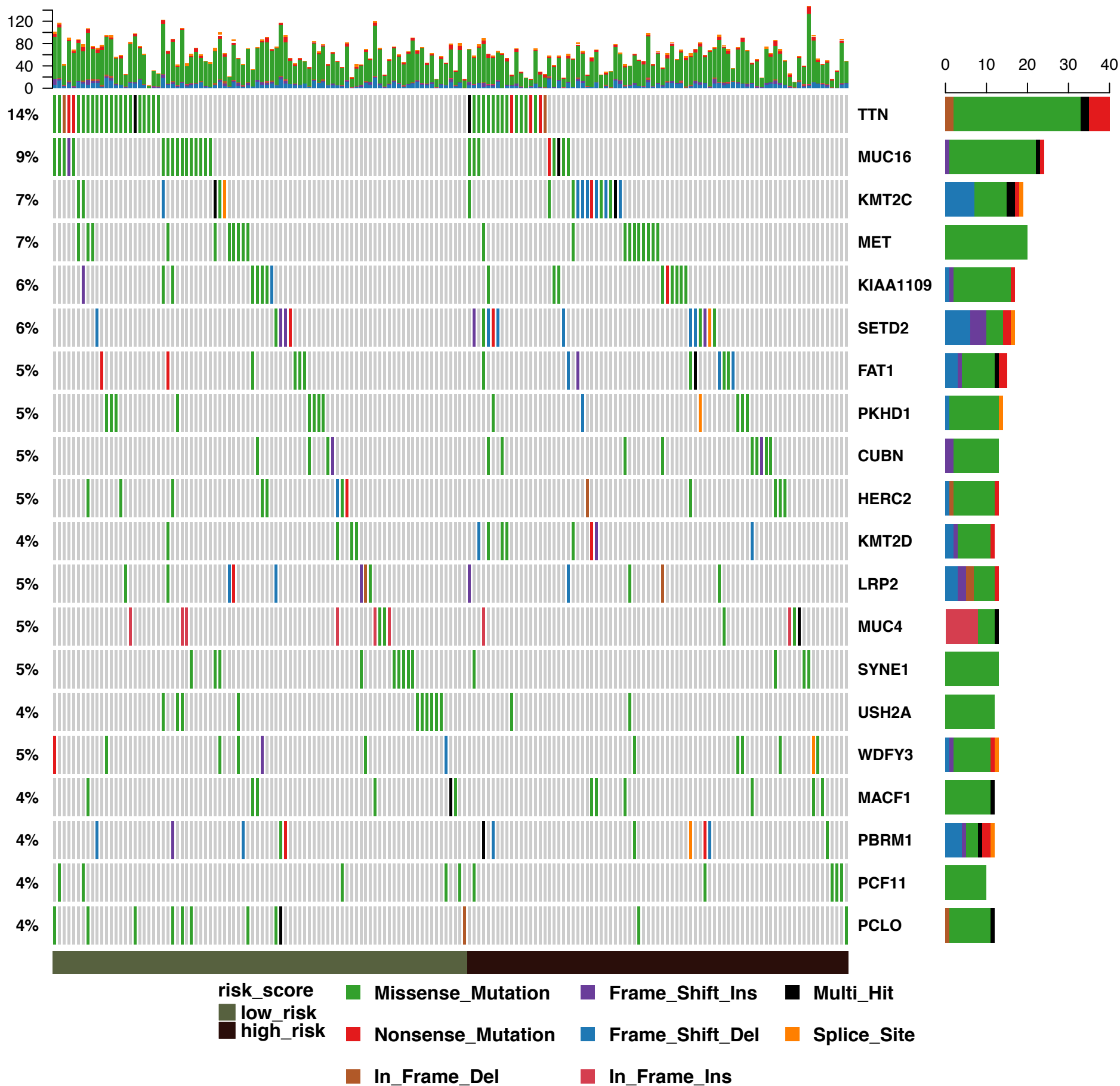

Supplement: Supplementary file 1 [file CAM4-8-289-s001.pdf]

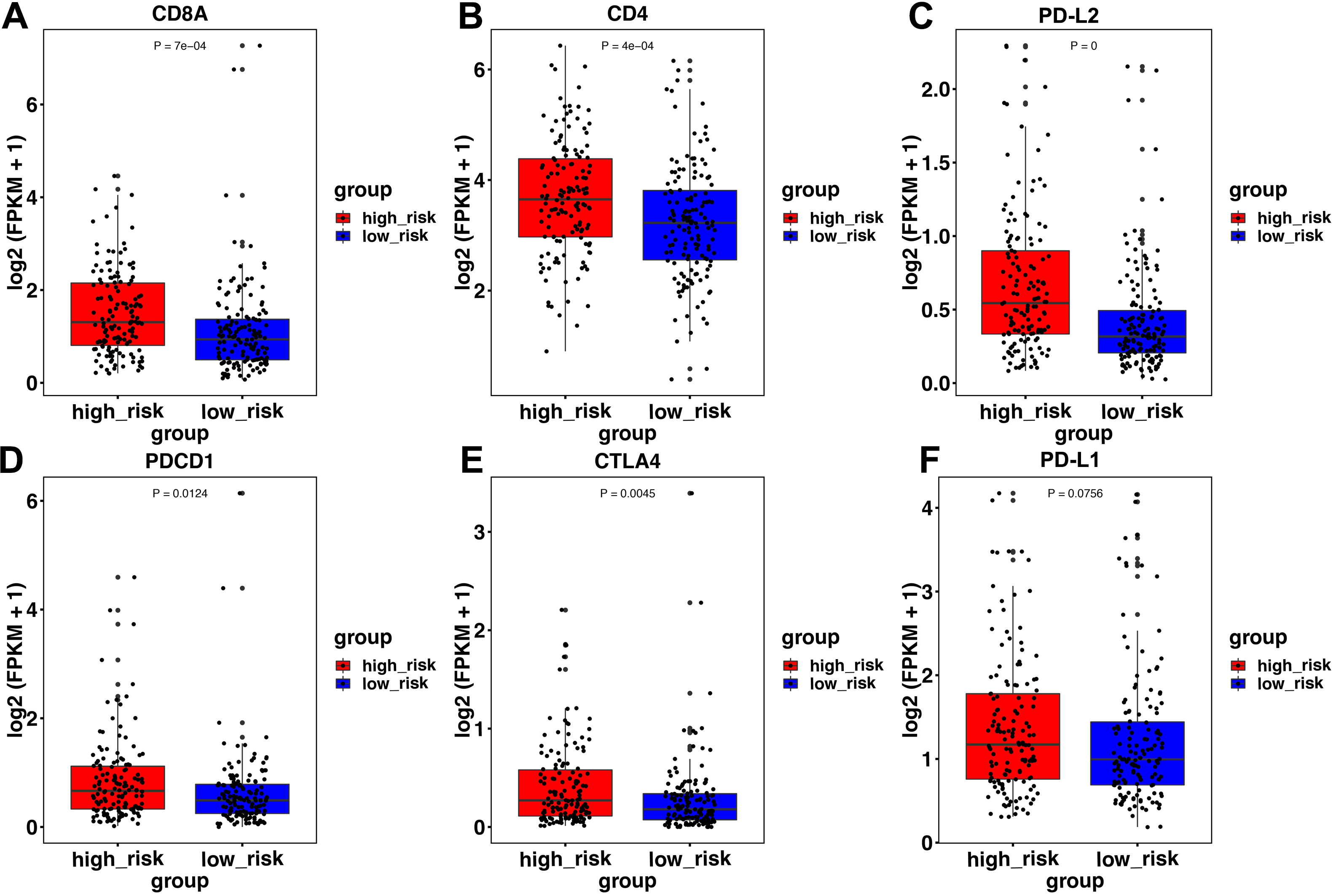

Supplement: Supplementary file 2 [file CAM4-8-289-s002.tif]
