# Supplementary material for: Construction of immune‐related risk signature for renal papillary cell carcinoma
Source: Cancer Med. 2018 Dec 5;8(1):289–304. doi: 10.1002/cam4.1905 (PMC6346237; doi:10.1002/cam4.1905)
Supplement: Supplementary file 3 [file CAM4-8-289-s003.docx]

| **Variable** | **Number of Patients**  **（n = 285）** | **Variable** | **Number of Patients**  **（n = 285）** |
| --- | --- | --- | --- |
| **Age** |  | **N stage** |  |
| ＞60 | 151 | N0 | 49 |
| ≤60 | 132 | N1 | 23 |
| NA | 2 | N2 | 4 |
| **Gender** |  | NX | 209 |
| Female | 76 | **M stage** |  |
| Male | 209 | M0 | 95 |
| **Tumor type** |  | M1 | 9 |
| Type 1 | 76 | MX | 181 |
| Type 2 | 84 | **Pathologic stage** |  |
| Unknown | 125 | Stage I | 170 |
| **Additional Therapy** |  | Stage II | 21 |
| YES | 24 | Stage III | 50 |
| NO | 261 | Stage IV | 15 |
| **T stage** |  | Stage X | 29 |
| T1 | 191 | **Recurrence** |  |
| T2 | 32 | YES | 145 |
| T3 | 58 | NO | 32 |
| T4 | 2 | Unknown | 108 |
| Tx | 2 |  |  |

Table S1: Summary of clinical and pathological features of KIRP patients
