# Supplementary material for: Construction of immune‐related risk signature for renal papillary cell carcinoma
Source: Cancer Med. 2018 Dec 5;8(1):289–304. doi: 10.1002/cam4.1905 (PMC6346237; doi:10.1002/cam4.1905)
Supplement: Supplementary file 6 [file CAM4-8-289-s006.docx]

| **Table S4. The relationship between the signature and staging and tumor type** | | | |
| --- | --- | --- | --- |
| **Variable** | **High risk**  **（n = 142）** | **Low risk**  **(n = 143)** | **P value** |
| **Tumor type** |  |  | 0.08 |
| Type 1 | 32 | 44 |  |
| Type 2 | 47 | 37 |  |
| **Pathologic T** |  |  | <0.001 |
| T1 | 78 | 113 |  |
| T2 | 18 | 14 |  |
| T3 | 44 | 14 |  |
| T4 | 2 | 0 |  |
| **Pathologic N** |  |  | <0.001 |
| N0 | 22 | 27 |  |
| N1-2 | 26 | 1 |  |
| **Pathologic M** |  |  | 0.01 |
| M0 | 53 | 42 |  |
| M1 | 9 | 0 |  |
| **Pathologic stage** |  |  | <0.001 |
| Stage I-II | 81 | 110 |  |
| Stage III-IV | 50 | 15 |  |

* Samples with NA or unknown value have been removed
