# Supplementary material for: Construction of immune‐related risk signature for renal papillary cell carcinoma
Source: Cancer Med. 2018 Dec 5;8(1):289–304. doi: 10.1002/cam4.1905 (PMC6346237; doi:10.1002/cam4.1905)
Supplement: Supplementary file 7 [file CAM4-8-289-s007.docx]

**Table S5:** Univariate Cox analysis of for overall survival of risk signature and clinical parameters in training, testing and all group after excluding cases with unknown T, N or M stage

|  | **Variable** | **HR** | **95%CI** | **pvalue** |
| --- | --- | --- | --- | --- |
| **Training**  **(n = 31)** | Risk score | 7.310 | 2.415-22.123 | <0.001 |
|  | age | 0.847 | 0.401-1.791 | 0.665 |
|  | stage (I and II vs III and IV) | 1.892 | 0.927-3.861 | 0.080 |
|  | T (T1 and T2 vs T3 and T4) | 1.637 | 0.423-6.332 | 0.475 |
|  | N (N0 vs N1 and N2) | 1.905 | 1.081-3.357 | 0.026 |
|  | M (M0 vs M1) | 3.630 | 1.655-7.959 | 0.001 |
|  | gender (male vs female) | 1.104 | 0.533-2.288 | 0.790 |
|  |  |  |  |  |
| **Testing**  **(n=14)** | Risk score | 1.383 | 0.615-3.111 | 0.433 |
|  | age | 0.492 | 0.209-1.160 | 0.105 |
|  | stage (I and II vs III and IV) | 3904.612 | 0-Inf | 0.999 |
|  | T (T1 and T2 vs T3 and T4) | 2.72E+08 | 0-Inf | 0.999 |
|  | N (N0 vs N1 and N2) | 1.771 | 0.734-4.275 | 0.204 |
|  | M (M0 vs M1) | 52421.57 | 0-Inf | 0.999 |
|  | gender (male vs female) | 1.671 | 0.601-4.648 | 0.325 |
|  |  |  |  |  |
|  | Risk score | 2.218 | 1.474-3.338 | < 0. 001 |
|  | age | 0.608 | 0.357-1.035 | 0.067 |
|  | stage (I and II vs III and IV) | 2.066 | 1.088-3.923 | 0.027 |
| **All**  **(n=45)** | T (T1 and T2 vs T3 and T4) | 2.436 | 0.823-7.21 | 0.108 |
|  | N (N0 vs N1 and N2) | 1.934 | 1.202-3.112 | 0.007 |
|  | M (M0 vs M1) | 5.248 | 2.279-12.084 | < 0.001 |
|  | gender (male vs female) | 1.207 | 0.675-2.158 | 0.526 |

Abbreviation: HR, hazard ratio; CI, confidence interval.
