# Supplementary material for: Construction of immune‐related risk signature for renal papillary cell carcinoma
Source: Cancer Med. 2018 Dec 5;8(1):289–304. doi: 10.1002/cam4.1905 (PMC6346237; doi:10.1002/cam4.1905)
Supplement: Supplementary file 8 [file CAM4-8-289-s008.docx]

| **Table S6:** Multivariate Cox analysis for overall survival of risk siganture and clinical parameters in training, testing and all group after excluding cases with unknown T, N or M stage | | | | |
| --- | --- | --- | --- | --- |
|  | **Variable** | **HR** | **95%CI** | **P value** |
| **Training**  **(n=31)** | Risk score | 27.981 | 1.176-665.596 | 0.039 |
|  | age | 2.504 | 0.288-21.757 | 0.405 |
|  | stage (I and II vs III and IV) | 5.747 | 0.111-296.628 | 0.385 |
|  | T (T1 and T2 vs T3 and T4) | 0.315 | 0.018-5.480 | 0.428 |
|  | N (N0 vs N1 and N2) | 0.100 | 0.001-6.956 | 0.287 |
|  | M (M0 vs M1) | 3.249 | 0.039-270.791 | 0.602 |
|  | gender (male vs female) | 0.476 | 0.046-4.948 | 0.534 |
|  |  |  |  |  |
| **Testing**  **(n=14)** | Risk score | 2.21E+107 | 0-Inf | 0.998 |
|  | age | 5.68E+25 | 0-Inf | 0.999 |
|  | stage (I and II vs III and IV) | 0 | 0-Inf | 0.998 |
|  | T (T1 and T2 vs T3 and T4) | 1 | 1-1 | NA |
|  | N (N0 vs N1 and N2) | 0 | 0-Inf | 0.998 |
|  | M (M0 vs M1) | Inf | 0-Inf | 0.998 |
|  | gender (male vs female) | 0 | 0-Inf | 0.999 |
|  |  |  |  |  |
| **All**  **(n=45)** | Risk score | 3.204 | 1.288-7.975 | 0.012 |
|  | age | 0.773 | 0.396-1.51 | 0.452 |
|  | stage (I and II vs III and IV) | 2.485 | 0.244-25.323 | 0.442 |
|  | T (T1 and T2 vs T3 and T4) | 0.509 | 0.079-3.282 | 0.478 |
|  | N (N0 vs N1 and N2) | 0.396 | 0.059-2.652 | 0.34 |
|  | M (M0 vs M1) | 85.655 | 4.635-1583.047 | 0.003 |
|  | gender (male vs female) | 2.397 | 0.259-22.189 | 0.441 |

Abbreviation: HR, hazard ratio; CI, confidence interval.
